# GPR105-Targeted Therapy Promotes Gout Resolution as a Switch Between NETosis and Apoptosis of Neutrophils

**DOI:** 10.3389/fimmu.2022.870183

**Published:** 2022-03-30

**Authors:** Chunxiao Liu, Mengze Zhou, Wenjiao Jiang, Shumin Ye, Sheng Tian, Cheng Jiang, Kun Hao, Huanqiu Li, Qinghua Hu

**Affiliations:** ^1^ State Key Laboratory of Natural Medicines, Key Laboratory of Drug Metabolism and Pharmacokinetics, China Pharmaceutical University, Nanjing, China; ^2^ School of Pharmacy, China Pharmaceutical University, Nanjing, China; ^3^ College of Pharmaceutical Sciences, Soochow University, Suzhou, China

**Keywords:** GPR105, NETosis, apoptosis, acute gouty arthritis, cAMP-PKA, lobetyolin

## Abstract

The fate of infiltrating neutrophils in inflamed joints determines the development of acute gouty arthritis (AGA). GPR105 highly expressed in human neutrophils is sensitive to monosodium urate crystals (MSU); nevertheless, the roles of GPR105 in AGA remain unclear. Here, we show that GPR105 is significantly upregulated in peripheral polymorphonuclear neutrophils of AGA patients. GPR105 knockout (GPR105^−/−^) prevented NETosis and induced apoptosis of neutrophils under MSU exposure, as well as attenuating inflammatory cascades in AGA. Mechanistically, GPR105 deletion activated cAMP-PKA signals, thereby disrupting Raf-Mek1/2-Erk1/2 pathway-mediated NADPH oxidase activation, contributing to inhibition of NETosis. Whereas, cAMP-PKA activation resulting in GPR105 deficiency modulated PI3K-Akt pathway to regulate apoptosis. More importantly, suppression of cAMP-PKA pathway by SQ22536 and H-89 restored NETosis instead of apoptosis in GPR105^−/−^ neutrophils, promoting MSU-induced gout flares. Interestingly, lobetyolin was screened out as a potent GPR105 antagonist using molecular docking-based virtual screening and *in vitro* activity test, which efficiently attenuated MSU-induced inflammatory response interacting with GPR105. Taken together, our study implicated that modulating cell death patterns between NETosis and apoptosis through targeting GPR105 could be a potential therapeutic strategy for the treatment of AGA.

## Introduction

Gout is a common arthritis caused by deposition of monosodium urate crystals (MSU) within joints under the condition of persistent hyperuricemia characterized by intense pain and swollen joints. When gout flares, MSU activates innate immune system and leads to inflammatory cascades accompanied by recruitment of neutrophils (1, 2).

As a key element of innate immune system, neutrophils are the first leukocytes to reach inflammatory sites during acute gouty arthritis (AGA), attempting to eliminate pathogens (3). MSU crystal-induced neutrophil activation leads to apoptosis inhibition and the formation of neutrophil extracellular traps (NETs), which was accompanied by degranulation, superoxide production, and cytokine release, subsequently expanding inflammatory reactions and leading to a characteristic cell death pattern of neutrophils called “NETosis” (4–6). Several studies have demonstrated that NETs could be separated from inflamed joints of patients with acute gout flares, which was composed by chromatin and granule constituents of neutrophils, indicating that NETosis might be the predominant cell death pattern in the pathogenesis of AGA (3, 7). Interestingly, neutrophils are demonstrated to be not required for resolution of AGA in mice, playing a detrimental role in the inflammatory phase of a gout attack (8). On the other hand, release of NETs might contribute to the formation of gouty tophus, which failed to be phagocytized by immune cells, leading to chronic gout (9). Therefore, inhibiting NETosis of neutrophils and promoting apoptosis of neutrophils should be an ideal strategy for treatment of AGA.

As a family of purinergic receptors, P2Y receptors (P2YRs) belong to the G protein-coupled receptor (GPCR) superfamily. Among them, GPR105, also known as P2Y_14_ receptor, could be activated by uridine diphosphate glucose sugars (UDP-sugars) to inhibit adenylate cyclase (AC)-mediated cAMP production *via* Gi/o-coupled protein (10), which was regarded to be involved in the modulation of immune inflammatory stress (11, 12). Recent studies proposed that MSU induced an upregulated GPR105 expression with a significant elevation in the secretion of inflammatory cytokines in human epidermal keratinocytes (NHEKs), intimating the roles of GPR105 in immune inflammatory responses exposed to MSU (13). Our previous study also demonstrates the role of GPR105 on pyroptosis of macrophages in the initial phase of AGA (14). However, how GPR105 intervenes in the inflammatory cascades of neutrophils remains undefined. Notably, PPTN, a specific antagonist of GPR105, inhibits UDPG-promoted chemotaxis of freshly isolated human neutrophils, indicating the functional expression of GPR105 in human neutrophils (15, 16). Since cAMP was illustrated to be related to cell death of neutrophils as a key secondary messenger (17–19), we investigated the role of GPR105 in pathogenesis of AGA through regulating cell death pattern of neutrophils centered on cAMP-PKA signals.

On the other hand, in order to develop potent and safe GPR105 antagonists for treatment of AGA, we tried to search a natural compound from the Natural Product Library with GPR105-binding capacity and GPR105 inhibitory activity through molecular docking-based virtual screening technology and *in vitro* bioassays. Furthermore, the effects of the candidate compounds on inflammatory response of neutrophils under MSU exposure were examined to evaluate the feasibility of GPR105-targeted therapy.

## Materials and Methods

### Reagent

All reagent and antibodies used are listed in **Supplementary Table S1**.

### Clinical Tissue Sample Collection and Ethic

All analyses were performed according to institutional guidelines with the approval of the ethical committee of Jiangsu Provincial Hospital of Traditional Chinese Medicine (2020NL-060-02). Informed consent was obtained from all subjects enrolled in the study. Neutrophils were isolated from heparinized blood of healthy volunteer and patients with acute gout flares or asymptomatic hyperuricemia by density gradient centrifugation and immediately stored at −80°C for assay. Total RNA was extracted from the neutrophils by Trizol Reagent and reverse transcribed to cDNA. Thereafter, real-time quantitative PCR was performed to analyze the gene expression levels. The primers are listed in **Supplementary Table S2**.

### Animals and Ethics

Heterozygous genotype GPR105^+/−^ Sprague Dawley (SD) rat was purchased from Beijing Biocytogen Co. Ltd. The offspring of homozygous (GPR105^−/−^) rat was obtained and identified for the following experiments. All animals were maintained in the animal facility at China Pharmaceutical University under SPF conditions. Genotypes were identified by PCR using specific primers (**Supplementary Figure S7**). All animal experiments were performed in conformity with the Guide for the Care and Use of Laboratory Animals (NIH publication No. 85-23, 1996 revision) and approved by the China Pharmaceutical University Committee for Laboratory Animal Use.

### Acute Gouty Arthritis Model

Male adult rats (8 weeks, 180~220 g) were randomized by weight and anesthetized with isoflurane and subjected to intra-articular injection with 1.5 mg of MSU crystals dissolved in 50 μl PBS in one ankle joint. PBS only was given to the contralateral ankle as a control. Intra-articular administration of forskolin (30 μM), bucladesine (300 μM), SQ22536 (100 μM), and H-89 (10 μM) was given to animals 30 min earlier before MSU injection. Ankle swelling was tested using a Vernier caliper.

### NETosis Assay

Primary neutrophils were plated at a density of 1 × 10^6^ per well in a 24-well plate precoated with 0.1 mg/ml poly-d-lysine in advance. After MSU stimulation (250 μg/ml) for 4 h, primary neutrophil was treated with SYTOX Green for monitoring NET release. NET release was then analyzed using Image J (NIH, Bethesda, MD, USA) as previously described and quantified with the positivity of SYTOX Green staining.

### Isolation and Purification of PMNs

Bone marrow-derived neutrophils were obtained from rat bone marrow by flushing femur and tibias using PBS. The cells were lysed with RBC lysis buffer and then subjected to density gradient centrifugation using Histopaque-1077 and Histopaque-1119 for 30 min at 1,000×*g*, 25°C. Primary neutrophils could be derived from the layer between Histopaque-1077 and Histopaque-1119. Neutrophil purity was verified by flow cytometry for CD11b and His48 (Beckton Dickinson, USA) (**Supplementary Figure S8**).

### Histopathological Studies of Synovium Tissues

The respective tissues were isolated and fixed overnight in buffered formalin 24 h after MSU injection. After being dehydrated, the synovium tissues were embedded in paraffin, sectioned for HE staining following standard protocols as previously described (14). The sections were observed under an IX53 microscope (Olympus, Japan) and photographed.

### Immunofluorescence

The sections of synovium tissue were deparaffinized with different grades of alcohol (100%–50%). A total of 1% Triton X-100 was used to permeabilized the tissues, and nonspecific binding sites were blocked with 5% goat serum for 1 h. Primary antibodies cit-H3-specific rabbit mAb (1:300) and MPO-specific mouse mAb (1:300) staining was then performed overnight at 4°C, followed by incubation with species-specific fluorogenic secondary antibodies for 2 h at room temperature. DAPI was used to mark the nucleus. For primary neutrophils, cells were fixed in 4% paraformaldehyde for 1 h and staining as described above. Immunofluorescent images were visualized by confocal laser scanning microscope (Zeiss LSM 700, Germany). The appropriate controls were maintained.

### Apoptosis Assay

Apoptosis in synovial tissues and primary neutrophils was measured by TUNEL assay staining according to manufacturer’s instructions. For synovial tissues, paraffin sections were prepared and stained with TUNEL reagents after sequential deparaffinization. For isolated neutrophils, cells were fixed in 4% paraformaldehyde with TUNEL reaction mixture at 37°C for 1 h.

### Western Blot

Protein lysates were extracted from neutrophils and synovium tissues by RIPA lysis buffer plus 10% PMSF. The protein lysates were boiled with 5× loading buffer and loaded in 10% or 15% SDS-PAGE to separate apart. The proteins were then transferred to PVDF membrane and analyzed by immunoblot with the appropriate antibodies as our previous study (14).

### ELISA

Neutrophils (1 × 10^6^/well) were seeded in 24-well plates and stimulated with 250 mg/ml MSU crystals for 4 h at 37°C. Levels of IL-8 and IL-1β in collected cell culture supernatants were detected using ELISA kits according to manufacturer’s protocol. Absorbance values were measured using a microplate reader (SpectraMax iD5, USA).

### Air-Pouch Model

Dorsal air pouches were prepared by injection of 30 ml sterile air on day 0. Three days after the first injection, 15 ml of sterile air were reinjected into the pouches. On day 7, MSU crystals (10 mg) in 2 ml PBS was injected into the air pouches and lavage was performed with 2 ml PBS at 24 h after MSU exposure. Air-pouch lavage fluids were centrifuged at 1,500 rpm for 5 min, and the supernatant was collected to quantify the NET release by SYTOX Green dye. Fluorescence (excitation, 488 nm; emission, 523 nm) was measured with a fluorescence microplate reader (SpectraMax iD5, USA). Cell pellet was suspended with PBS, and neutrophils were isolated by density gradient centrifugation. Purified neutrophils were used for Western blot test.

### Glide Docking-Based Virtual Screening Pipeline

Two well-established GPR105 homology models (HM1 and HM2) (20) proposed by Trujillo et al. were selected and minimized for the following docking-based virtual screening pipeline. Three scoring functions of Glide docking (HTVS, SP XP) were applied to perform the sequential VS strategy. The 300 highest-ranked compounds of the prepared Natural Compound Library (more than 2,000 compounds) predicted by HTVS were redocked using SP scoring mode. The 300 highest-ranked compounds of SP were then recalculated using the XP scoring function. Finally, 100 highest-ranked compounds were obtained for each GPR105 homology model. The compounds with promising drug-like properties were then reserved by removing duplicates, applying Lipinski’s “Rule-of-Five” filter, the REOS criteria, and drug-likeness models built in our previous studies. The compounds with less than two chiral centers were then retained and the remaining compounds were clustered using the Tanimoto coefficient evaluated based on MACCS structural keys (Tanimoto coefficient cutoff value = 0.7). Lastly, 10 compounds were selected for experimental testing.

### GPR105 Inhibitory Activity Screening

HEK293-hGPR105 cells obtained from Keygen Biotech were grown to 85%–90% confluency prior to assays in DMEM supplemented with FBS. Cells were stimulated with 30 μM forskolin followed by 10 μM UDPG, and then the cells underwent treatment for 0.5 h with various concentrations of test compounds. The cAMP levels were then detected *via* cAMP-Glo™ Assay. IC_50_ value was calculated by SPSS.

### Statistical Analysis

Values for all figures refer to mean ± SD. Results were analyzed and compared by one-way ANOVA. Differences were regarded as statistical significance once *p*-value was <0.05.

## Results

### GPR105 Functions as a Switch From Apoptosis to NETosis in MSU-Induced Acute Inflammation

**Figure 1:**
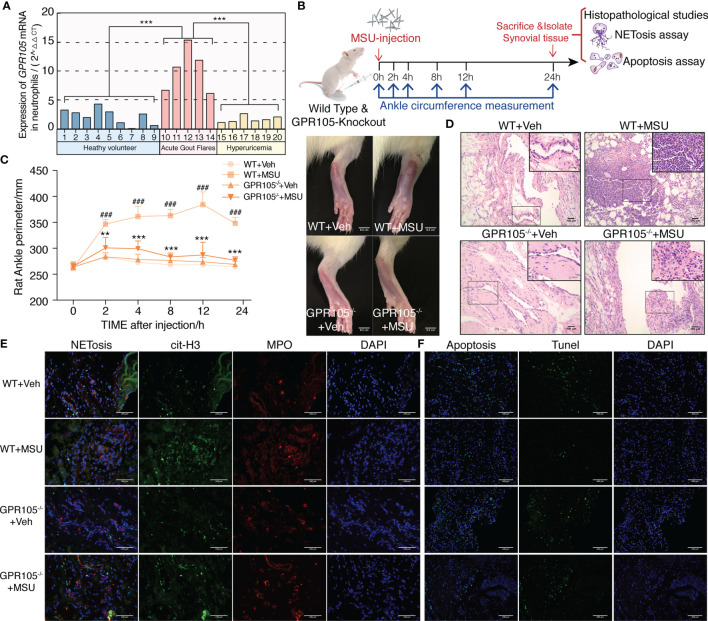
GPR105^−/−^ suppresses MSU-induced acute gouty inflammation. **(A)** Expression of GPR105 mRNA in neutrophils isolated from heathy volunteers (*n* = 9), acute gout flare patients (*n* = 5), and asymptomatic hyperuricemia patients (*n* = 6) was quantified using qPCR. Gene expression for each volunteer is independently represented as a bar. **(B)** GPR105^−/−^ and WT rats were intra-articularly injected with vehicle (Veh) or MSU (5 mg/ml) to induce acute gouty arthritis (*n* = 4) (^###^ *p*  <  0.001 versus corresponding WT+Veh group. ^**^ *p* < 0.01; ^***^ *p* < 0.001 versus corresponding WT+MSU group, one-way ANOVA). **(C)** The ankle perimeter at 0, 2, 4, 8 12, and 24 h after MSU injection. The representative photograph of ankle at 8 h after MSU injection. **(D)** Histologic analyses of synovial tissues at 24 h after MSU injection. **(E)** Representative images of synovial tissues infiltrating neutrophils (MPO, red) and presence of NETs (cit-H3, green). **(F)** Representative images of apoptosis cell (TUNEL, green) in synovial tissues.

As shown in **Figure 1A**, the mRNA level of GPR105 in neutrophils of AGA patients was significantly higher than those of asymptomatic hyperuricemia patients and healthy volunteers, indicating that GPR105 might play an important role in the pathogenesis of acute gout flares.

**Figure 2:**
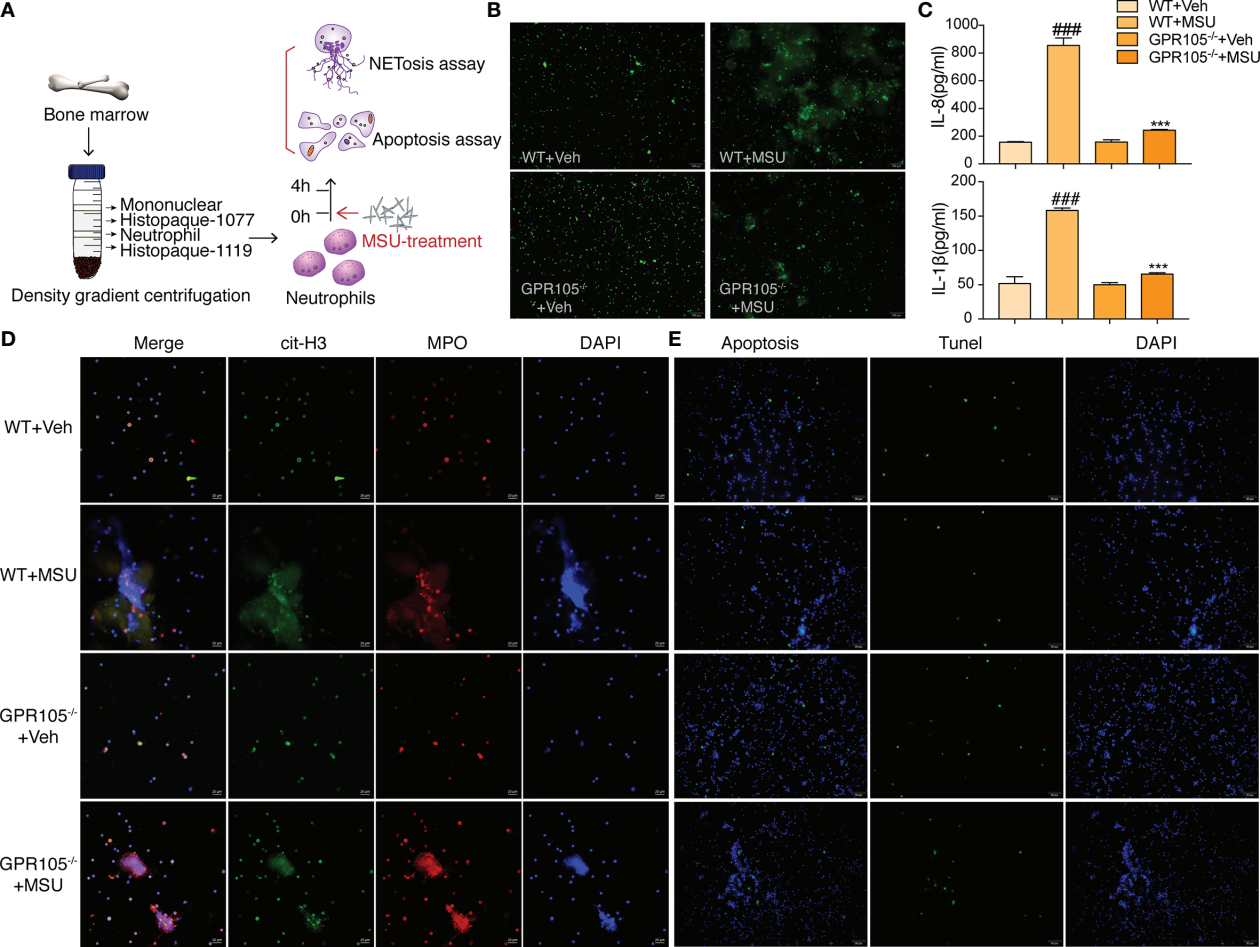
GPR105^−/−^ suppresses MSU-induced NETosis. **(A)** Primary neutrophils purified from WT and GPR105^−/−^ rats were incubated with Veh or MSU (250 μg/ml) for 4 h (*n* = 3). **(B)** Representative images of extracellular DNA staining (SYTOX green) in primary neutrophils. **(C)** Levels of IL-8 and IL-1β in the MSU-treated neutrophil supernatant were measured by ELISA. All values are presented as the mean ± SD (^###^ *p* < 0.001 versus corresponding WT+Veh group. ^***^ *p* < 0.001 versus corresponding WT+MSU group, one-way ANOVA). **(D)** Representative images of NET formation (citrullinated H3, green; MPO, red) in primary neutrophils. Representative images of apoptosis cell (TUNEL, green) in primary neutrophils. **(E)** Representative images of apoptosis cell (TUNEL, green) in primary Neutrophils.

Next, AGA model was established in wild-type (WT) and GPR105 knockout (GPR105^−/−^) rats to explore the role of GPR105 in acute gouty inflammation. **Figures 1B–D** shows that the joint swelling and inflammatory infiltration induced by MSU injection decreased in GPR105^−/−^ rats. In addition, we noted that ablation of GPR105 significantly attenuated MSU-induced NET formation according to the results of the colocalization between citrullinated histone H3 (cit-H3) and MPO (**Figure 1E**). Similar to the results observed in rats with AGA, knockout of GPR105 also suppressed NET release in MSU-induced air pouch model as the result of SYTOX Green staining (**Supplementary Figure S1C**). Consistent with the results observed *in vivo*, NET formation and secretion of IL-8 and IL-1β in GPR105^−/−^ neutrophils decreased significantly under MSU stress, when compared with WT neutrophils (**Figures 2A–D**).

On the other hand, TUNEL data revealed that a significant upregulation occurs in the rate of apoptosis in MSU-stimulated synovium of GPR105^−/−^ rats in contrast with those of WT rats (**Figure 1F** and **Supplementary Figure S1A**). In *in vitro* studies, GPR105 knockout raised the apoptotic level in PMNs under MSU stimulation (**Figure 2E** and **Supplementary Figure S1B**).

### cAMP-PKA Pathway Is Involved in the Mechanism Responsible for Switch Role of GPR105 in Conversion Between NETosis and Apoptosis

**Figure 3:**
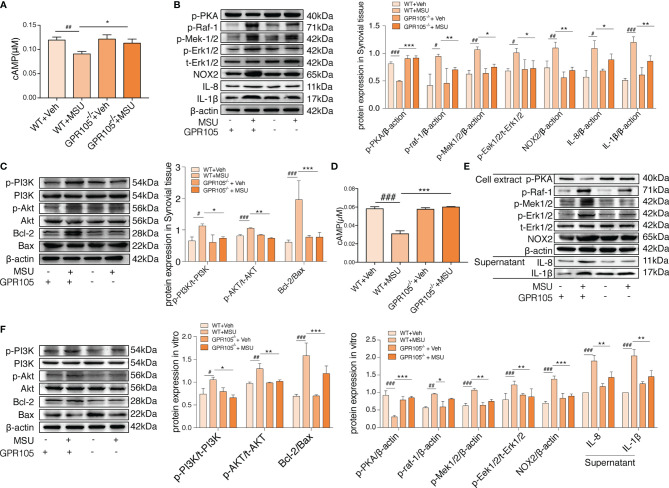
cAMP-PKA pathways play a key role in GPR105-regulated acute inflammation and conversion between NETosis and apoptosis. **(A)** The concentration of intracellular cAMP in synovial tissue of WT or GPR105^−/−^ rats 24 h after Veh or MSU (5 mg/ml) injected (*n* = 4). **(B)** Western blot analysis and relative quantification of NETosis-related proteins in synovial tissues. **(C)** Western blot analysis and relative quantification of apoptosis-related proteins in synovial tissues. **(D)** The concentration of intracellular cAMP of primary neutrophils purified from WT or GPR105^−/−^ rats 4 h after incubating with Veh or MSU (250 μg/ml) (*n* = 3). **(E)** Western blot analysis and relative quantification of NETosis-related proteins in primary neutrophils. Quantification of the protein level of IL-1β and IL-8 in supernatant was expressed as the ratio of WT+vehicle group. **(F)** Western blot analysis and relative quantification of apoptosis-related proteins in primary neutrophils. All representative blots shown are from four independent experiments. All values are presented as the mean ± SD (^#^ *p* < 0.05; ^##^ *p* < 0.01; ^###^ *p* < 0.001 versus corresponding WT+Veh group. ^*^ *p* < 0.05; ^**^ *p* < 0.01; ^***^ *p* < 0.001 versus corresponding WT+MSU group, one-way ANOVA).

Given the fact that GPR105 is a Gi-coupled receptor, we hypothesized that GPR105 knockdown might have an impact on the production of downstream mediator cAMP. As shown in **Figure 3A**, GPR105 knockout resulted in a significant elevation in the cAMP level of synovial when compared with WT ones under MSU conditions. Consistent with cAMP alteration, the expression of p-PKA decreased after MSU injection, which was prevented upon deficiency of GPR105 (**Figure 3B**). Additionally, similar changes were observed in the p-PKA expression of neutrophils derived from air-pouch model (**Supplementary Figure S1D**). More importantly, like the results observed *in vivo*, ablation of GPR105 also restored the expression of cAMP and p-PKA in MSU-stimulated primary neutrophils (**Figures 3D, E** **)**, suggesting that cAMP-PKA might play an essential role in GPR105-mediated NETosis under MSU stress.

With regard to NETosis, the Raf/Mek/Erk pathway has been implicated in NET formation through NOX2 activation (21), while extracellular signaling of UDP-glucose induces phosphorylation of ERK1/2 MAP kinases in HL-60 cell (22). We assessed the activation status of Raf/Mek/Erk and the expression of NOX2 to further explore mechanisms of the effect of GPR105 on MSU-induced NETosis. As **Figure 3B** shows, the synovial expression of p-Raf-1, p-Mek1/2, p-Erk1/2, and NOX2 markedly increased after MSU exposure in WT rats, which was unobservable in MSU-treated GPR105^−/−^ rats. Also, the neutrophils of the air-pouch model exhibited a similar phenomenon in Raf/Mek/Erk signaling (**Supplementary Figure S1D**). Meanwhile, the activation of Raf/Mek/Erk was also suppressed by knockout of GPR105 in MSU-treated primary neutrophils (**Figure 3E**).

Next, to get a better comprehension on the role of GPR105 in apoptosis, the expression of apoptosis-related proteins would be evaluated. Since Akt is reported to be involved in the switch between NADPH-dependent NETosis and apoptosis (23), we examined the activation of PI3K/Akt and the ratio of Bcl-2 to Bax, which had been widely reported in apoptosis signaling. Western blot analysis showed that the activation of PI3K/Akt pathway hoisted significantly after MSU injection in synovium samples of WT rats but not in GPR105^−/−^ rats. Meanwhile, the analysis of Bal-2/Bax confirmed a lesser apoptosis rate in MSU-induced WT rats compared with GPR105^−/−^ rats (**Figure 3C**). The apoptotic-related protein expression of neutrophils in air-pouch model was consistent with those of synovial tissues in gouty arthritis model (**Supplementary Figure S1E**). These results also corresponds with the Western blot analysis result of PI3K/Akt/Bcl-2/Bax pathway in MSU-stimulated primary neutrophils (**Figure 3F**), indicating that GPR105 possessed the effects on MSU-induced apoptosis by regulating PI3K/Akt/Bcl-2/Bax pathway.

We also investigated whether the upregulation of cAMP-PKA pathway could alleviate MSU-induced acute inflammation. As expected, activated cAMP-PKA signals by treatment with forskolin and bucladesine both suppressed MSU-induced NETosis together with the downregulation of Raf-1/Mek/Erk pathway. MSU-mediated apoptosis was delayed, and PI3K/Akt pathway activation was effectively suppressed by forskolin and bucladesine administration (**Supplementary Figure S2**). These results were mirrored in MSU-induced air-pouch model and MSU-stimulated primary neutrophils (**Supplementary Figures S3, S4**).

### Inhibited cAMP-PKA Abolishes the Protective Effect of GPR105 Knockout in Rats

**Figure 4:**
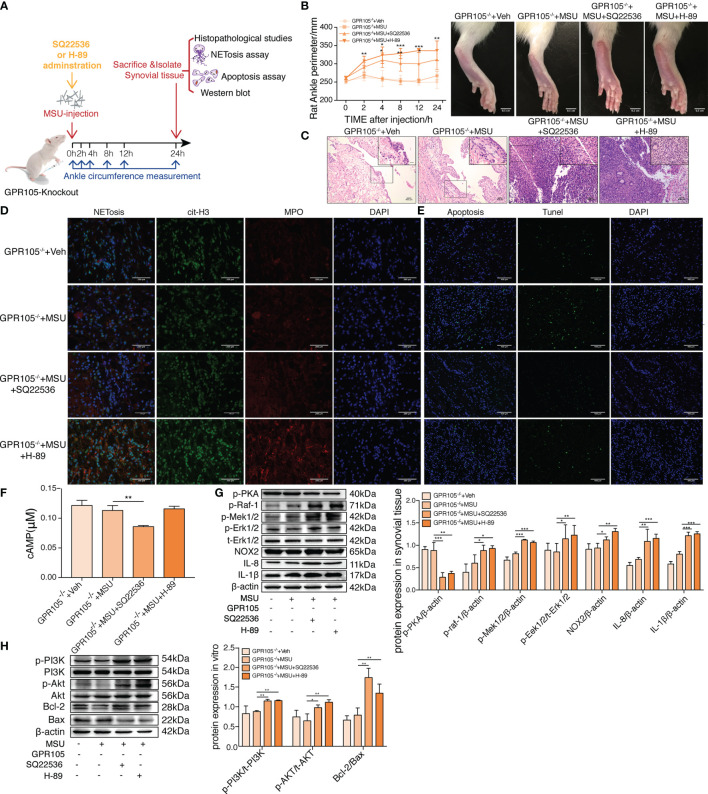
Inhibition of cAMP-PKA pathway suppresses the effect of GPR105 knockout and switches apoptosis to NETosis *in vivo.* **(A)** GPR105^−/−^ rats were intra-articularly injected with 5 mg/ml MSU to induce gouty arthritis. Rats were additionally treated with SQ22536 (100 μM), H-89 (10 μM), or DMSO as respectively indicated (*n* = 4). **(B)** The ankle perimeter at 0, 2, 4, 8 12, and 24 h after MSU injection. The representative photograph of ankle at 8 h after the injection of MSU. **(C)** Histologic analyses of synovial tissues at 24 h after MSU injection. **(D)** Representative images of synovial tissues infiltrating neutrophils (MPO, red) and presence of NETs (cit-H3, green). **(E)** Representative images of apoptosis cell (TUNEL, green) in synovial tissues. **(F)** The concentration of intracellular cAMP in synovial tissue of GPR105^−/−^ rats 24 h after intra-articularly injected with Veh or MSU (5 mg/ml). **(G)** Western blot analysis and relative quantification of NETosis-related proteins in synovial tissues. **(H)** Western blot analysis and relative quantification of apoptosis-related proteins in synovial tissues. All representative blots are shown from four independent experiments. All values are presented as the mean ± SD (^*^ *p* < 0.05; ^**^ *p* < 0.01; ^***^ *p* < 0.001 versus corresponding GPR105^−/−^+MSU group, one-way ANOVA).

To prove our hypothesis that cAMP-PKA could be responsible for the effect of GPR105 on two programmed cell death, SQ22536 (inhibitor of AC) and H-89 (inhibitor of PKA) treatment would be given to GPR105^−/−^ rats to inhibit cAMP-PKA pathway (**Figure 4F**). In rat gouty arthritis model, it could be observed that SQ22536 and H-89 treatment exhibited a significant exacerbation of ankle swelling and neutrophil infiltration into synovium (**Figures 4A–C**). Confocal microscope images showed that administration of SQ22536 and H-89 reversed MSU tolerance of GPR105^−/−^ with a significant increment of NETosis (**Figure 4D**). In consistent with exaggerated NETosis, Western blot presented a markedly provoked IL-1β, IL-8, NOX2, and Raf/Mek/Erk pathway in SQ22536 and H-89 groups, suggesting that inhibited cAMP-PKA exaggerated MSU-induced NETosis *in vivo* (**Figure 4G**). Meantime, the decrease in TUNEL-positive cells and in the expression of PI3K/AKT/Bcl-2/Bax pathway in SQ22536 groups and H-89 groups also supported this hypothesis, suggesting that inhibited cAMP-PKA lead to a suppressed apoptosis with an enhanced NETosis (**Figures 4E, H**; **Supplementary Figure S5A**). Similarly, SQ22536 and H-89 also inhibited apoptosis and promoted NETosis in MSU-induced air-pouch model (**Supplementary Figure S5C–E**).

### cAMP-PKA Inhibition Disrupts the Resistance of GPR105 Knockout in Primary Neutrophils

**Figure 5:**
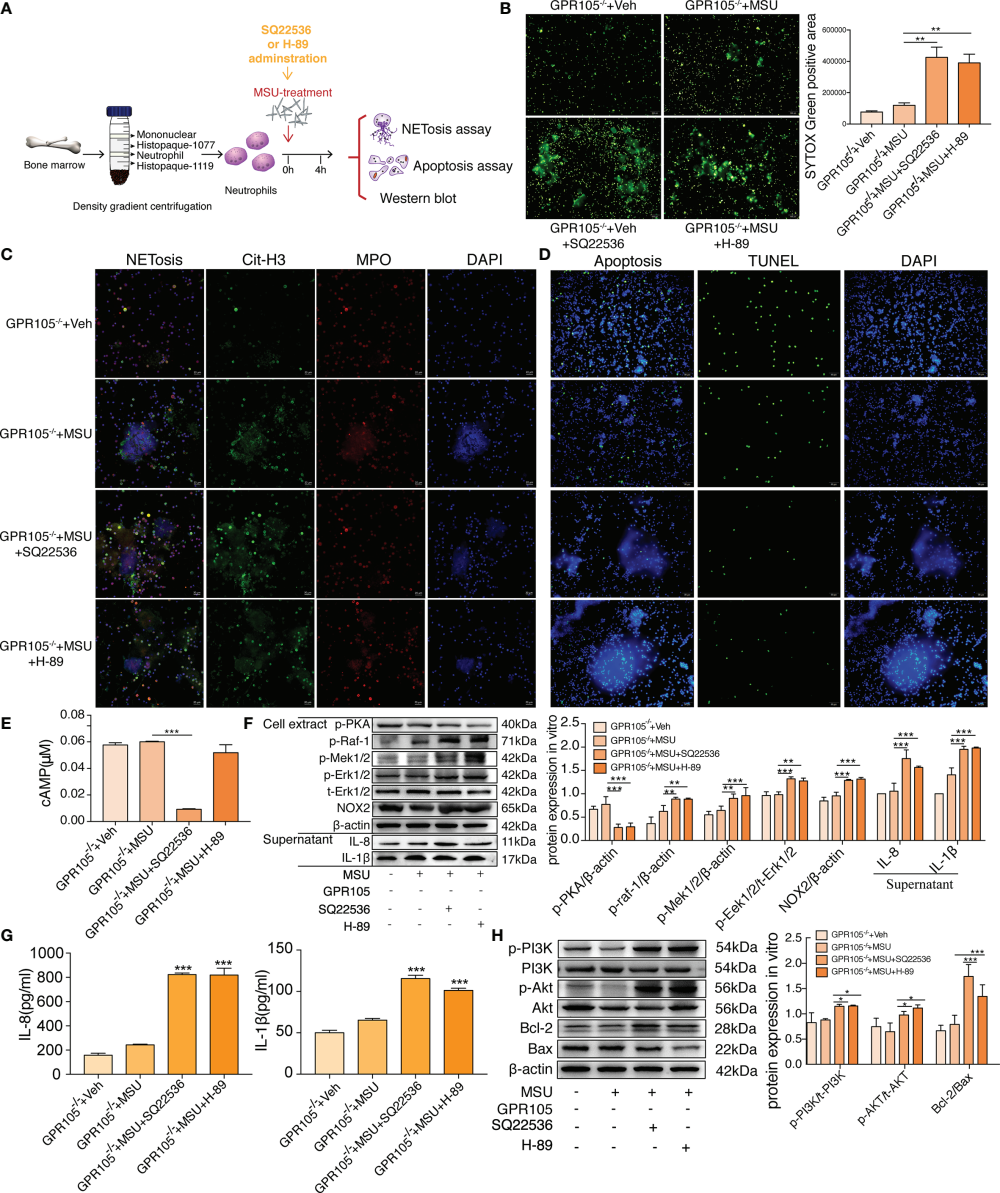
Inhibition of cAMP-PKA pathway suppresses the effect of GPR105 knockout and switches apoptosis to NETosis *in vitro*. **(A)** Primary neutrophils were prepared from the bone marrow of GPR105^−/−^ rats and then treated with 250 μg/ml MSU. Neutrophils were additionally incubated with SQ22536 (100 μM), H-89 (10 μM), or Veh as respectively indicated (*n* = 3). **(B)** Representative images of extracellular DNA staining (SYTOX green, green) in primary neutrophils. **(C)** Representative microphotographs of NET formation (citr-H3, green and MPO, red) in primary neutrophils. **(D)** Representative images of apoptosis cell (TUNEL, green) in synovial tissues. **(E)** Intracellular concentration of cAMP in primary neutrophils. **(F)** Western blot analysis and relative quantification of NETosis-related proteins in primary neutrophils. Quantification of the protein level of IL-1β and IL-8 in supernatant was expressed as the ratio of GPR105^−/−^ +Veh group. **(G)** Levels of IL-8 and IL-1β in the MSU-treated neutrophil supernatant were measured by ELISA. **(H)** Western blot analysis and relative quantification of apoptosis-related proteins in primary neutrophils. All representative blots are shown from three independent experiments. All values are presented as the mean ± SD (^*^ *p* < 0.05; ^**^ *p* < 0.01; ^***^ *p* < 0.001 versus corresponding GPR105^−/−^+MSU group, one-way ANOVA).

As expected, incubation with SQ22536 and H-89 significantly neutralized the protective effect of GPR105 knockout on MSU-induced NETosis and cytokine release in GPR105^−/−^ neutrophils (**Figures 5A–C, G** **)**. Analysis of Western blot showed that the activation of Raf/Mek/Erk pathway and the expression of NOX2 all increased apparently in the SQ22536 and H-89 groups, compared with the MSU group, indicating that inhibited cAMP-PKA caused by SQ22536 and H-89 completely abolished the GPR105-mediated tolerance of MSU-induced NETosis (**Figures 5E, F** **)**. Next, we examined the rate of apoptosis in MSU-stimulated GPR105^−/−^ neutrophils after treatment of SQ22536 and H-89 and observed that SQ22536 and H-89 decreased the apoptosis rate and disrupted the promotive effect of GPR105 knockout on apoptosis, which was confirmed by a notable upregulation of PI3K/Akt/Bcl-2/Bax in GPR105^−/−^ neutrophils (**Figures 5D, H** and **Supplementary Figure S5B**).

### Molecular Docking-Based Virtual Screening and GPR105 Inhibitory Activity Screening

**Figure 6:**
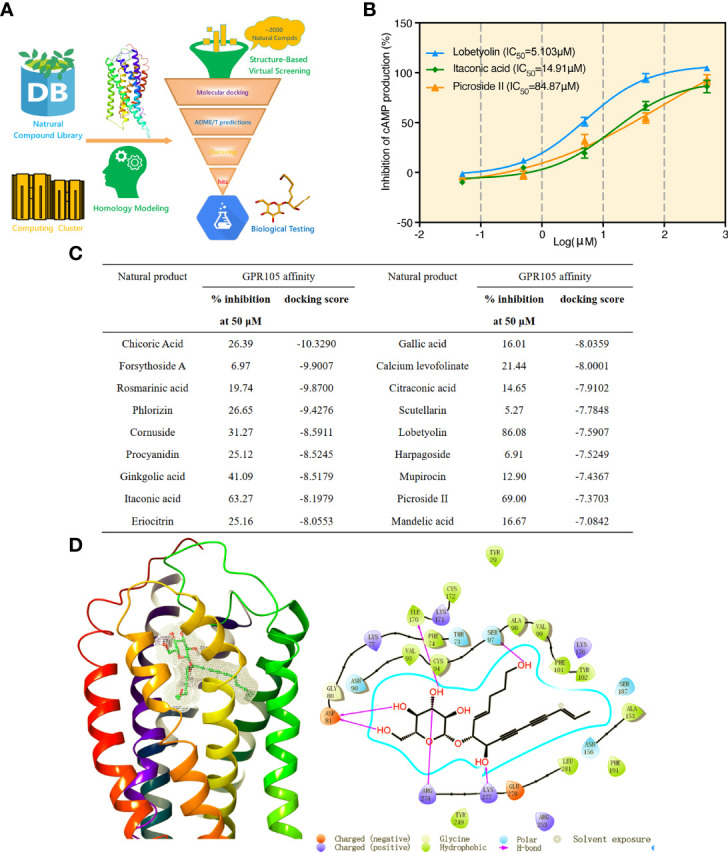
Molecular docking-based virtual screening and GPR105 inhibitory activity screening. **(A)** Workflow of the structure-based virtual screening. **(B)** Luminescence assay of GPR105-binding affinities (IC50 curves) of three identified GPR105 antagonists (lobetyolin, itaconic acid, and picroside II). **(C)** Biological activities and key parameters identified in docking-based VS of the 18 purchased compounds from the Natural Compound Library. **(D)** The predicted binding pose of lobetyolin and the interaction patterns between lobetyolin and key residues in the binding site of GPR105 from Glide docking.

We used Glide docking pipeline to screen potential GPR105 antagonists from the Natural Compound Library (2,592 natural compounds), More detailed information of Glide docking pipeline can be seen in our previous study (24). Followed by ADME/T predictions and structural clustering, 18 natural compounds were eventually purchased for biological testing (**Figure 6A**). Furthermore, we determined GPR105 inhibitory activities of selected compounds based on the production of cAMP in HEK293-hGPR105 cells. As the result presented in **Figure 6C**, 3 of 18 purchased compounds (VS hit rate = 16.67%) showed quite acceptable inhibitory activities against GPR105. The IC_50_ value of identified GPR105 antagonists is shown in **Figure 6B**. Among them, lobetyolin (LBT) showed the most potent antagonistic activity (IC_50_ = 5.103 μM). Thus, we further examined the inhibition of LBT in MSU-induced neutrophil NETosis *in vitro* and observed that low or medium concentration (10 μM, 50 μM) of LBT could markedly suppress MSU-induced NET formation in neutrophils (**Supplementary Figure S6A**).

In addition, the binding pose predicted by Glide docking for LBT and key interaction patterns between LBT and GPR105 were briefly highlighted and analyzed. **Figure 6D** shows that LBT could form stable interactions (hydrogen bonds) with favorable residues including Asp81, Ser97, Ile170, Arg274, and Lys277 in the binding pocket of GPR105.

### Lobetyolin Inhibition MSU-Induced AGA *In Vivo* and *In Vitro*

**Figure 7:**
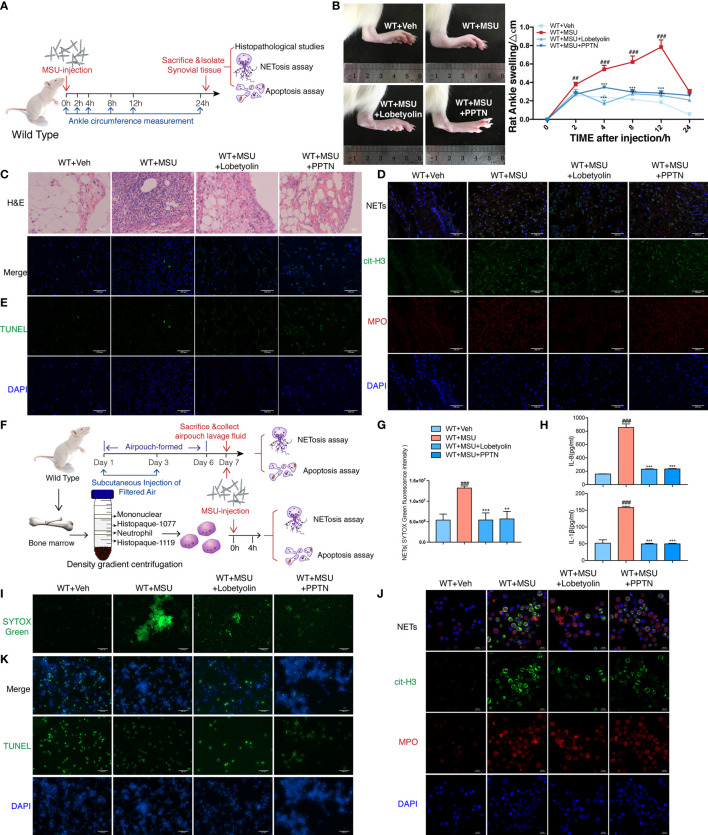
Lobetyolin inhibits MSU-induced acute gouty arthritis *in vivo* and *in vitro*. **(A)** WT rats were intra-articularly injected with 5 mg/ml MSU to induce gouty arthritis. Rats were additionally treated with lobetyolin (LBT) (10 μM) and PPTN (10 μM) or Veh as respectively indicated (*n* = 4). **(B)** The ankle perimeter at 0, 2, 4, 8 12, and 24 h after MSU injection. The representative photograph of ankle at 8 h after the MSU injection. **(C)** Histologic analyses of synovial membrane. **(D)** Representative images of synovial tissues infiltrating neutrophils (MPO, red) and presence of NETs (cit-H3, green). **(E)** Representative images of apoptosis cell (TUNEL, green) in synovial tissues. **(F)** Primary neutrophils were treated with 250 μg/ml MSU (*n* = 3). Neutrophils were additionally incubated with LBT (10 μM), PPTN (10 μM), or Veh as respectively indicated. **(G)** Sytox green assay was used to quantify neutrophil extracellular DNA release in air pouches. **(H)** Levels of IL-8 and IL-1β in the MSU-treated neutrophil supernatant were measured by ELISA. **(I)** Representative images of extracellular DNA staining (SYTOX green) in neutrophils. **(J)** Representative microphotographs of NET formation (cit-H3, green and MPO, red) in neutrophils. **(K)** Representative images of apoptosis cell (TUNEL, green) in neutrophils. All values are presented as the mean ± SD (^##^ *p* < 0.01; ^###^ *p* < 0.001 versus corresponding WT+Veh group. ^**^ *p* < 0.01; ^***^ *p* < 0.001 versus corresponding GPR105^−/−^+MSU group, one-way ANOVA).

To assess the pharmacological effect of LBT in MSU-induced AGA *in vivo*, we established gouty arthritis model in WT rats; LBT and PPTN were injected into ankle joints simultaneously with MSU. We found that LBT mitigated ankle swelling and neutrophil infiltration in synovium. More importantly, LBT reduced NETosis and promoted apoptosis of neutrophils in synovial tissues accompanied with expected regulated effects on Raf-Mek1/2-Erk1/2-NOX2 and PI3K-Akt signaling pathways, which were consistent with findings in MSU-mediated rat air-pouch model. Moreover, these *in vivo* results were mirrored in MSU-stimulated purified bone marrow neutrophils. In summary, these data demonstrated that LBT could suppress MSU-induced acute inflammation and mediate conversion of neutrophil death pattern *via* targeting GPR105 (**Figure 7** and **Supplementary Figure S6B–G**).

## Discussion

Acute gout arthritis is a prevailing and incapacitating disease resulting from deposition of MSU in joints, characterized by the recruitment of neutrophils into the sites of inflammation (25). Recently, it has been demonstrated that neutrophils act as a proinflammatory factor in the early phase of AGA in animal models. Moreover, neutrophil-depleted mice exhibited similar patterns of tissue swelling diminution in the resolution phase (8), indicating that neutrophil activation by MSU played a detrimental effect during bouts of AGA.

Crystal-induced neutrophil activation leads to the formation of NETs, which is present in effusion from acutely inflamed joints of gout patients consistent with DNA, contributing to the formation of gouty tophi as the core component (3, 9). On the other hand, neutrophil apoptosis and uptake of apoptotic material by macrophages drive the resolution of acute inflammation (26). Therefore, imbalance of the cell death patterns between NETosis and apoptosis might contribute to the development of AGA with unclear mechanisms. Consistently, reduction of NETs release using P2Y_6_R antagonist (MRS2578) could apparently reverse inflammatory reactions induced by MSU (27). In addition, ROCK inhibition by treatment with Y-27632 as well as blockade of PI3Kγ or PI3Kδ could resolve acute inflammation by enhancing neutrophil apoptosis in a murine model of gout (28, 29). In our present study, GPR105 deficiency significantly attenuated acute inflammatory cascades induced by MSU through promoting neutrophil to undergo apoptosis rather than NETosis, which further verified feasibility of the therapeutic strategy targeting neutrophils.

Extracellular nucleotides secreted from various tissues or organs play indispensable roles in regulating innate and adaptive immune responses as potent ligands through activating respective purinergic receptors (30). Several recent studies revealed that GPR105 acted as an essential factor in acute inflammatory responses under the condition of glycogen metabolism dysregulation (31), which had been regarded as a potential target for the prevention and/or attenuation of ischemic-AKI (32, 33). Our previous studies also demonstrated that novel GPR105 antagonists could effectively improve MSU-induced AGA in cell models (24, 34), implying the role of GPR105 in mediating immune inflammation. The present study verified the overexpression of GPR105 in peripheral polymorphonuclear neutrophils of gout patients during an acute stage, which provided clinical evidences for our research. Furthermore, GPR105 activated by UDP-sugars was upregulated after allergen challenge in neutrophils (35), while UDP-glucose effectively promoted chemotaxis of freshly prepared human neutrophils (15). Interestingly, our findings firstly proved that GPR105 was closely allied to cell death pattern of neutrophils after MSU stimulation, disturbing the immune inflammatory response of neutrophils during acute gout flares.

cAMP-PKA signaling pathways may represent a therapeutic target for the abrogation of neutrophil dysfunction in various inflammatory diseases (17, 36). Exogenous PGE2 administration decreased NETosis in an exchange protein activated by cAMP/PKA-dependent manner (37). The AC toxin inhibits formation of NETs by producing cAMP, suppressing the oxidative burst (38). Moreover, NETosis induced by assembly of the NADPH machinery could be decreased after pentoxifylline (PTX) treatment, which was reversed by pretreatment of PKA inhibitor H-89 through Raf-Mek-Erk pathways. Consistently, our present study exhibited that forskolin or bucladesine activated PKA and interfered phosphorylation of Raf-1, Mek1/2, and Erk1/2, subsequently restraining NOX2-dependent NETosis. Meanwhile, inhibitory effects of GPR105 knockout on MSU-induced NETosis in AGA was diminished by the presence of SQ22536 and H89, suggesting a cAMP-PKA-dependent mechanism. On the other hand, neutrophils with downregulated cAMP were defective in their ability to undergo apoptosis, leading to impaired clearance of neutrophils from the inflamed joint and failed arthritis resolution (19). In addition, PDE4 inhibition resolved neutrophilic inflammation through accelerating apoptosis of inflammatory cells, which was dependent on PKA/PI3K/Akt-signals (39). As expected, delayed apoptosis of neutrophils induced by MSU was restored by induction of AC or PKA activator, while antiapoptotic effect attributing to the lack of GPR105 was abrogated by blocking cAMP-PKA signals.

An air-pouch model in rats, considered a useful model of inflammation, is commonly applied for studies on neutrophilic inflammatory diseases (40). MSU induces a significant increase in pouch fluid leukocytes at several hours, which has been regarded as an ideal method for studying immune function of neutrophils (41). GPR105 knockout abolished NETosis and accelerated apoptosis of neutrophils recruited into air pouches with MSU treatment. Next, the cAMP-PKA signals were concerned about the mediators between GPR105 and cell death patterns of neutrophils *via* Raf-Mek-Erk and PI3K-Akt pathways.

LBT, the critical ingredient extracted from *Codonopsis pilosula*, which is a famous traditional Chinese medicine, has been reported to exert numerous biological effects, such as anticancer, anti-inflammatory, and antioxidative and xanthine oxidase-inhibiting properties (42–44). However, the effect of LBT in MSU-induced AGA remains to be under investigation. In the current work, LBT has been found to be a potent GPR105 antagonists according to the results of cascade docking-based VS and antagonistic activity testing. Thus, we further investigated the effect of LBT on an MSU-induced AGA and neutrophil death pattern. We established MSU-induced AGA model, air-pouch model, and *in vitro* gout model of neutrophils, revealing that LBT could suppress MSU-induced AGA by inhibiting the inflammatory activities of neutrophils. LBT-treated rats exhibited apparent reductions in joint swelling and leukocyte recruitment. More importantly, our results suggested that LBT inhibited neutrophil NETosis and promoted apoptosis under MSU conditions *in vivo* and *in vitro.* In addition, the dominant favorable residues of LBT interacting with GPR105 are Asp81, Ser97, Ile170, Arg274, and Lys277. These findings indicated that LBT might be an available therapeutic agent for treatment of AGA by mediating the neutrophil death pattern through targeting GPR105. However, several studies have reported that LBT has antioxidant effects, and ROS plays an important role in the occurrence of NETosis. As a natural compound, LBT may also inhibit NETosis through other mechanisms, including antioxidation. Further studies are needed to explore the mechanisms by which LBT inhibits NETosis.

**Figure 8:**
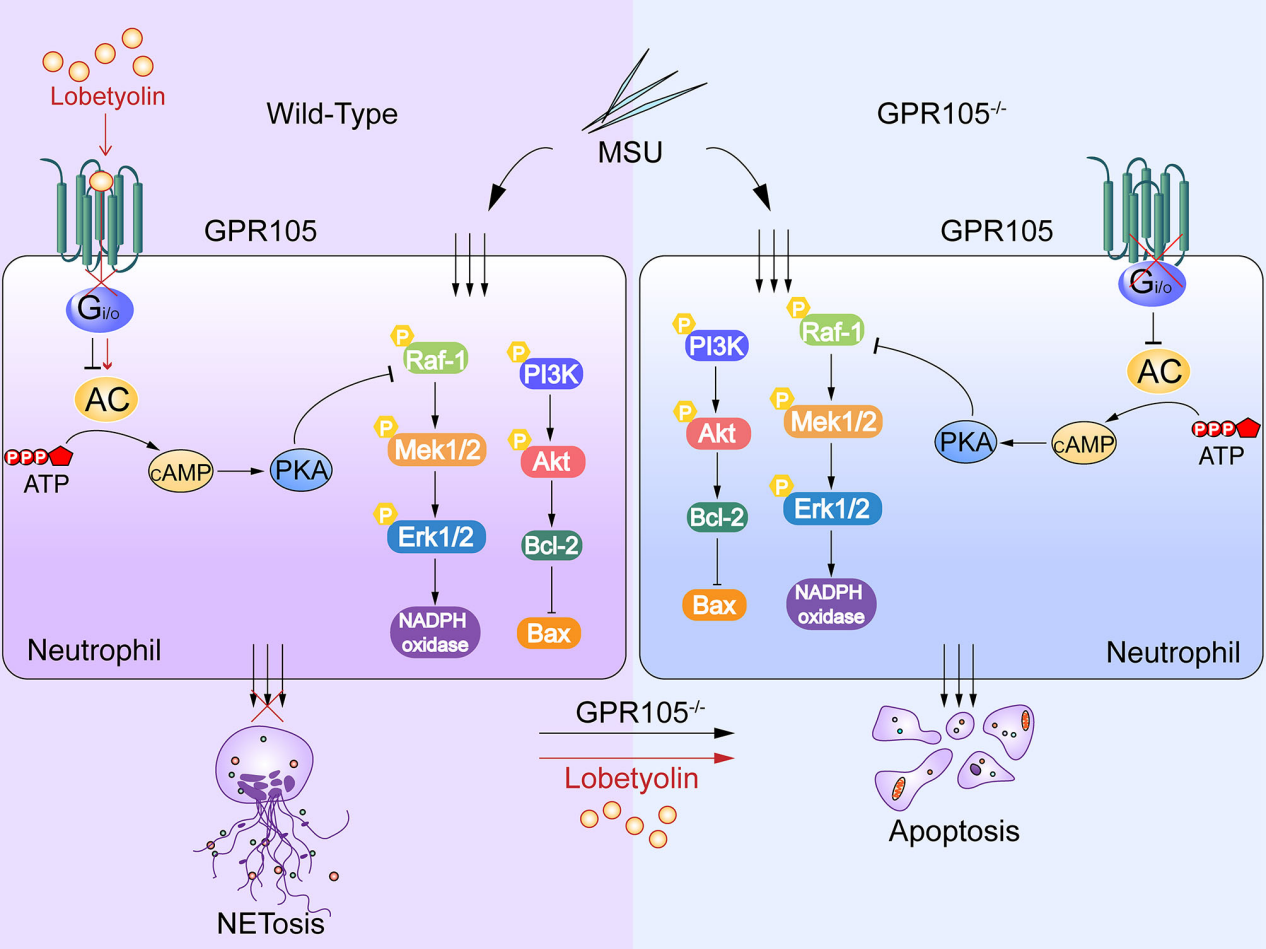
MSU induces NETosis and inhibits apoptosis in neutrophils. Lobetyolin suppresses MSU-induced acute gouty arthritis and mediates conversation of neutrophil death pattern *via* targeting GPR105.

In summary, the present study demonstrated the causal role of GPR105 in AGA as a switch between NETosis and apoptosis in neutrophils. cAMP-PKA signaling pathways were proposed to mediate crosstalk between GPR105 activation and cell death patterns of neutrophils in acute gout flares. In addition, LBT as a GPR105 antagonist could be a potential AGA therapeutic drug. Nevertheless, further investigation is necessary to verify the interaction of GPR105 and LBT at the molecular level (**Figure 8**).

## Data Availability Statement

The raw data supporting the conclusions of this article will be made available by the authors, without undue reservation.

## Ethics Statement

The animal study was reviewed and approved by The China Pharmaceutical University Committee for Laboratory Animal Use. Written informed consent was obtained from the individual(s) for the publication of any potentially identifiable images or data included in this article.

## Author Contributions

QH did the concept and study design. KH participate in the pharmacodynamic experimental design for lobetyolin. CL and MZ performed the experiments. ST and HL did the molecular docking-based virtual screening. SY and CJ did the statistical analysis and interpreted the results. WJ drafted the manuscript. QH and HL revised the manuscript. All authors reviewed and commented on the manuscript and approved its final submission.

## Funding

This research was supported by the National Natural Science Foundation of China (Grants 81773745, 81872867, 82073685) and Natural Science Foundation of Jiangsu Province (BK20211223).

## Conflict of Interest

The authors declare that the research was conducted in the absence of any commercial or financial relationships that could be construed as a potential conflict of interest.

## Publisher’s Note

All claims expressed in this article are solely those of the authors and do not necessarily represent those of their affiliated organizations, or those of the publisher, the editors and the reviewers. Any product that may be evaluated in this article, or claim that may be made by its manufacturer, is not guaranteed or endorsed by the publisher.

## Abbreviations

AC, adenylate cyclase; AGA, acute gouty arthritis; AID, autoinflammatory disease; MSU, monosodium urate; NETs, neutrophil extracellular traps; UDPG, uridine diphosphate glucose; LBT, lobetyolin.
