# Supplementary material for: GPR105-Targeted Therapy Promotes Gout Resolution as a Switch Between NETosis and Apoptosis of Neutrophils
Source: Front Immunol. 2022 Mar 30;13:870183. doi: 10.3389/fimmu.2022.870183 (PMC9005804; doi:10.3389/fimmu.2022.870183)
Supplement: Supplementary file 1 [file DataSheet_1.pdf]

Supplementary Materials for

GPR105-targeted therapy promotes gout resolution as a switch between NETosis and apoptosis of neutrophils

Chunxiao Liu, Mengze Zhou, Wenjiao Jiang, Shumin Ye, Sheng Tian, Cheng Jiang, Hao Kun, Huanqiu Li, Qinghua Hu

Correspondence to: huqh@cpu.edu.cn

This PDF file includes:

Supplementary Figures 1 to 8 and Supplementary Tables 1,2

Supplementary Figures

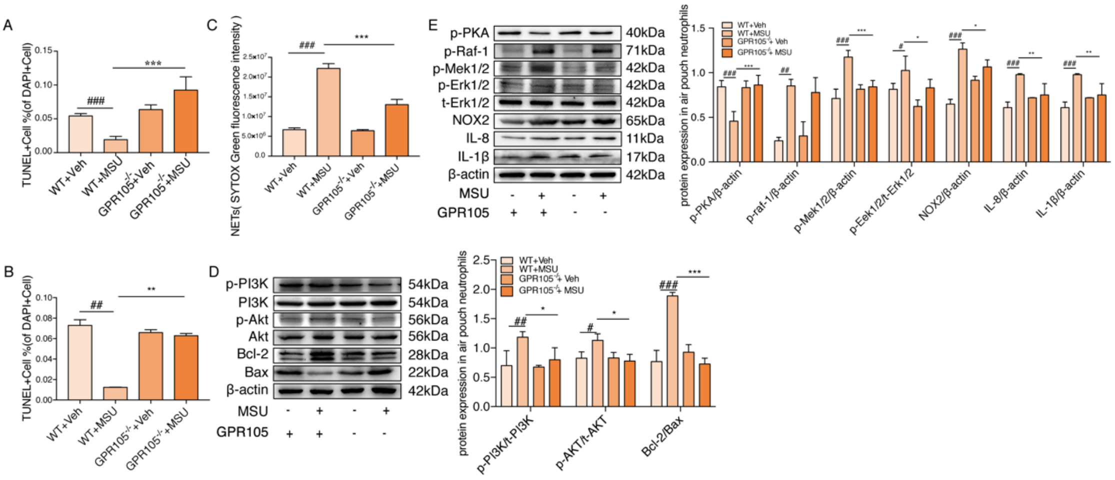

Supplementary Fig. 1 GPR105$^{-/-}$ suppresses MSU-induced acute gouty inflammation, related to Figures 1 and 2. A Quantitative analysis of TUNEL-positive cells in Figure 1**F**. The apoptotic index was calculated as the number of TUNEL-positive cells/DAPI positive cells. **B** Quantitative analysis of TUNEL-positive cells in Figure 2**E**. The apoptotic index was calculated as the number of TUNEL-positive cells/DAPI positive cells. For **C** to **D**, GPR105$^{-/-}$ and WT rats were injected with 5 mg/ml MSU into pre-formed air pouches on the backs. **C** Sytox green assay was used to quantify neutrophil extracellular DNA release in air pouches (n=4). **D** Western blot analysis and relative quantification of apoptosis-related proteins in air pouch neutrophils using the indicated antibodies. **E** Western blot analysis and relative quantification of NETosis-related proteins in air pouch neutrophils using the indicated antibodies. All representative blots shown are from four independent experiments. All values are presented as the mean ± SD ($^{\#}P < 0.05$, $^{\#\#}P < 0.01$, $^{\#\#\#}P < 0.001$ versus corresponding WT+Vehicle group. $^{*}P$<0.05; $^{**}P$<0.01; $^{***}P$<0.001 versus corresponding WT+MSU group, One–Way ANOVA).

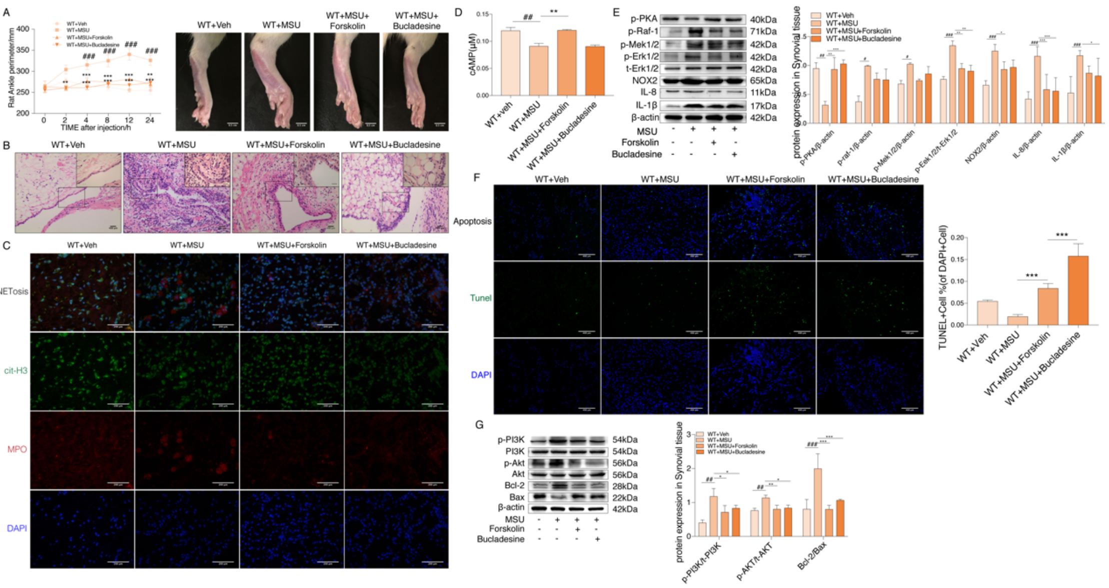

Supplementary Fig. 2 Activation of cAMP-PKA pathway suppresses NETosis and promotes apoptosis *in vivo*. WT rats were intra-articularly injected with 5 mg/ml MSU crystals to induce gouty arthritis. Rats were additionally treated with Forskolin (30 μM), Bucladesine (300μM) or Veh respectively as indicated. **A** The ankle perimeter at 0, 2, 4, 8 12, 24 h after MSU injection. The representative photograph of ankle at 8 h after injection of MSU. **B** Histologic analyses of synovial membrane at 24 h after MSU injected. The small boxed areas show higher magnification views of the large boxes. **C** Representative images of synovial tissues infiltrating neutrophils (MPO, red) and presence of NETs (citrullinated H3, green). **D** Intracellular cAMP levels of synovial tissue. **E** Western blot analysis and relative quantification of NETosis-related proteins in synovial tissues using the indicated antibodies. **F** Representative images of apoptosis cell (TUNEL, green) in synovial tissues. The apoptotic index was calculated as the number of TUNEL-positive cells/DAPI positive cells. **G** Western blot analysis and relative quantification of apoptosis-related proteins in synovial tissues using the indicated antibodies. All representative blots shown are from four independent experiments. All values are presented as the mean ± SD ($^{\#}P < 0.05$, $^{\#\#}P < 0.01$,

$^{###}P<0.001$ versus corresponding WT+Vehicle group. $^{*}P<0.05$; $^{**}P<0.01$; $^{***}P<0.001$ versus corresponding WT+MSU group, One–Way ANOVA).

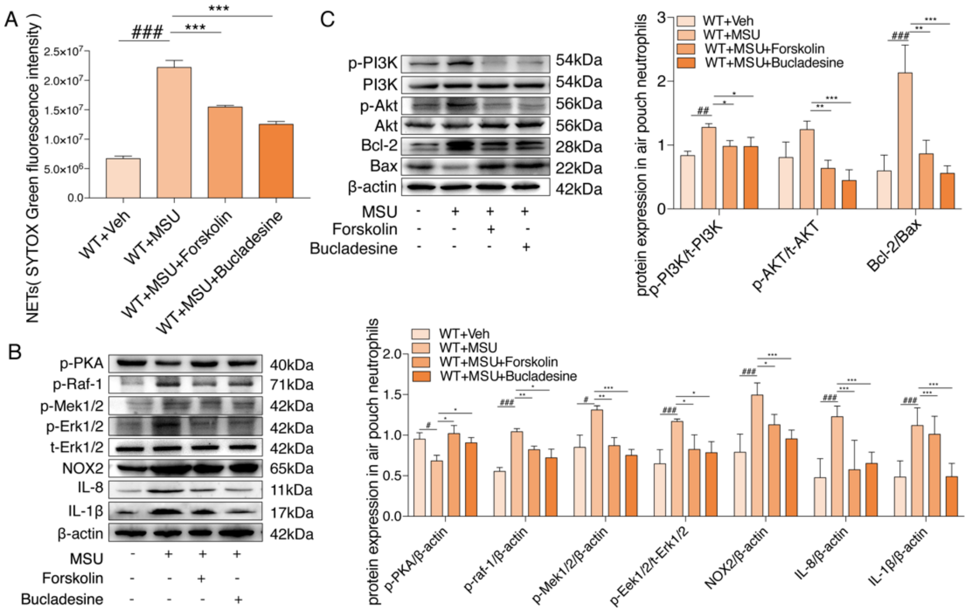

Supplementary Fig. 3 Activation of cAMP-PKA pathway suppresses NETosis and promotes apoptosis in air pouch model. WT rats were injected with 5 mg/ml MSU into pre-formed air pouches on the backs. Rats were additionally treated with Forskolin (30 μM), Bucladesine (300μM) or Veh respectively as indicated. **A** Sytox green assay was used to quantify neutrophil extracellular DNA release in air pouches 24 h after injection of MSU crystals in WT rats. **B** Western blot analysis and relative quantification of NETosis-related proteins in air pouch neutrophils using the indicated antibodies 24 h after MSU injected into pre-formed air pouches. **C** Western blot analysis and relative quantification of apoptosis-related proteins in air pouch neutrophils using the indicated antibodies 24 h after MSU injected into pre-formed air pouches. All representative blots shown are from four independent experiments. All values are presented as the mean ± SD. ($^{\#}P<0.05$, $^{\#\#}P<0.01$, $^{###}P<0.001$ versus corresponding WT+Vehicle group. $^{*}P<0.05$; $^{**}P<0.01$; $^{***}P<0.001$ versus corresponding WT+MSU group, One–Way ANOVA).

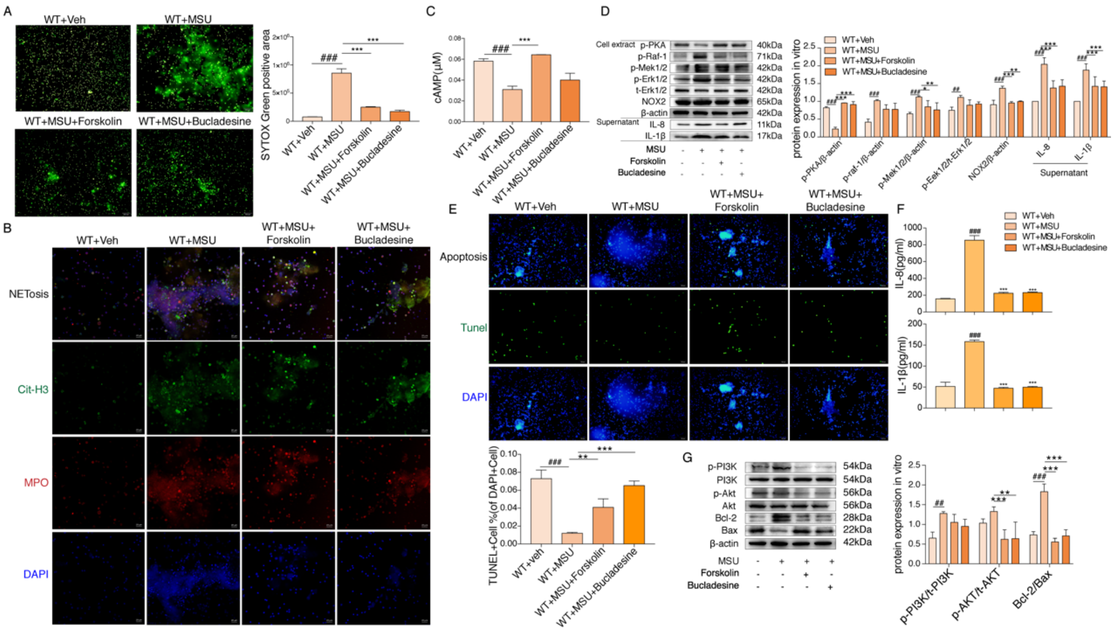

Supplementary Fig. 4 Activation of cAMP-PKA pathway suppresses NETosis and promotes apoptosis *in vitro*. Primary Neutrophils were treated with 250 μg/ml MSU. Primary Neutrophils were additionally incubated with Forskolin (30 μM), Bucladesine (300 μM) or Veh respectively as indicated (n=3). **A** Representative microphotographs and relative quantification of extracellular DNA staining (SYTOX green, green) in primary Neutrophils. **B** Representative microphotographs of NETs formation (citrullinated H3, green and MPO, red) in primary Neutrophils. **C** Intracellular concentration of cAMP in primary neutrophils. **D** Western blot analysis and relative quantification of NETosis-related proteins in primary neutrophils using the indicated antibodies. Quantification of the protein level of IL-1β and IL-8 in supernatant was expressed as the ratio of WT+Vehicle group. **E** Representative images of apoptosis cell (TUNEL, green) in synovial tissues. The apoptotic index was calculated as the number of TUNEL-positive cells/DAPI positive cells. **F** Levels of IL-8 and IL-1β in the MSU-treated neutrophils supernatant measured by ELISA. **G** Western blot analysis and relative quantification of apoptosis-related proteins in primary neutrophils using the indicated antibodies. All representative blots shown are from three independent experiments. All

values are presented as the mean ± SD ($^{\#}P < 0.05$, $^{\#\#}P < 0.01$, $^{\#\#\#}P < 0.001$ versus corresponding WT+Vehicle group. $^{*}P$<0.05; $^{**}P$<0.01; $^{***}P$<0.001 versus corresponding WT+MSU group, One–Way ANOVA).

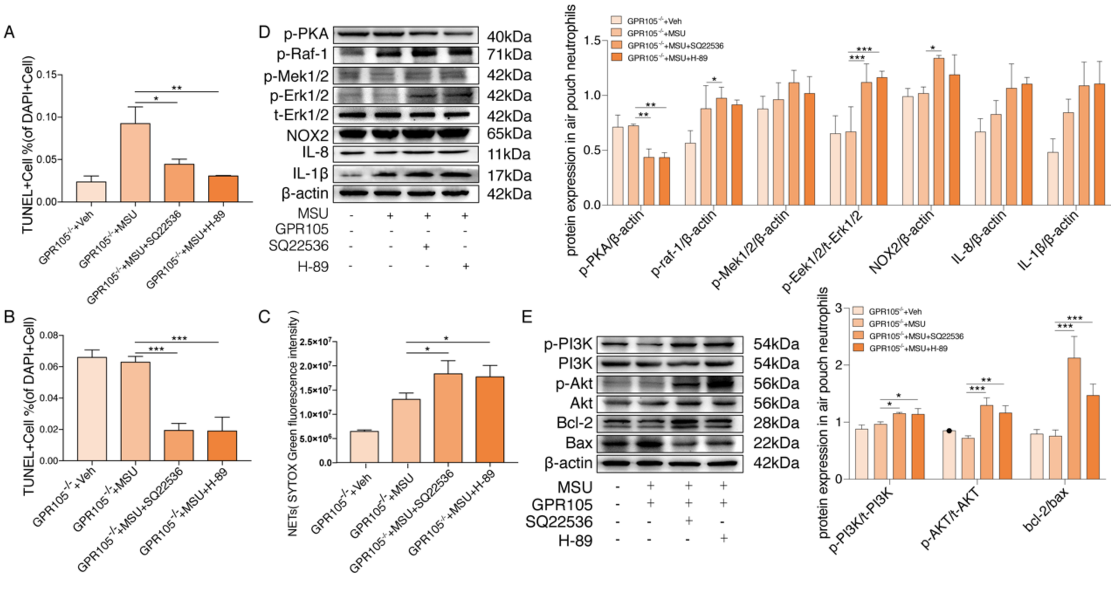

Supplementary Fig. 5 Inhibition of cAMP-PKA pathway suppresses the effect of GPR105 knockout and switches apoptosis to NETosis, related to Figures 3 and 4. A and **B**, Quantitative analysis of TUNEL-positive cells in Fig. 4**E** and Fig. 5**D**. The apoptotic index was calculated as the number of TUNEL-positive cells/DAPI positive cells. For **C** to **E**, GPR105$^{-/-}$ rats were injected with 5 mg/ml MSU into pre-formed air pouches on the backs. Rats were additionally treated with SQ22536 (100 μM), H-89 (10 μM) or DMSO respectively as indicated. **C** Sytox green assay was used to quantify neutrophil extracellular DNA release in air pouches 24 h after injection of MSU crystals in WT rats. **D** Western blot analysis and relative quantification of NETosis-related proteins in air pouch neutrophils using the indicated antibodies 24 h after MSU crystals injected into pre-formed air pouches. **E** Western blot analysis and relative quantification of apoptosis-related proteins in air pouch neutrophils using the indicated antibodies 24 h after MSU crystals injected into pre-

formed air pouches. All representative blots shown are from three independent experiments. All values are presented as the mean ± SD ($^{*}P$<0.05; $^{**}P$<0.01; $^{***}P$<0.001 versus corresponding GPR105$^{-/-}$+MSU group, One–Way ANOVA).

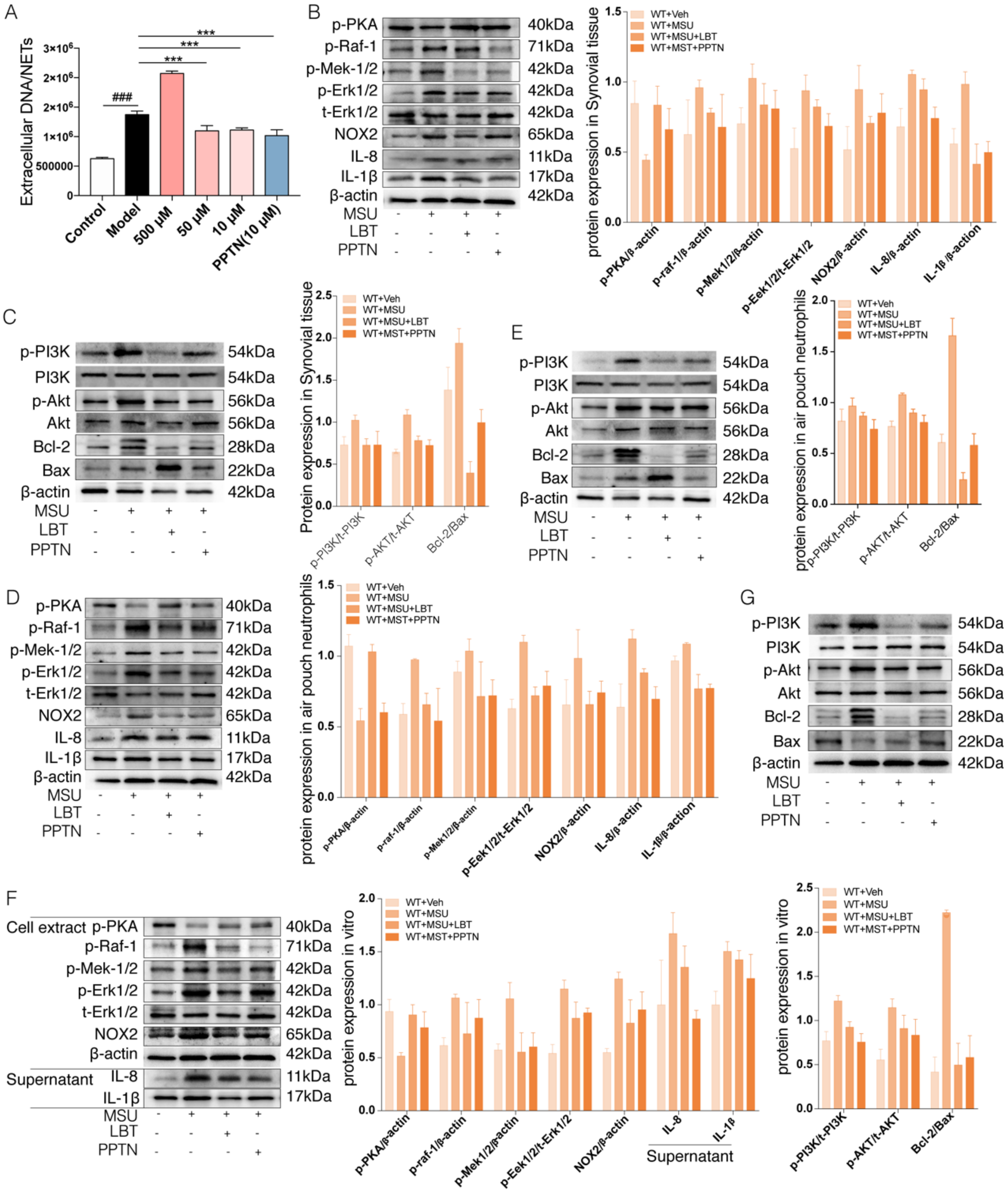

Supplementary Fig. 6 LBT inhibition MSU-induced acute gouty arthritis in vivo and in vitro, related to Figures 5 and 6. A Effects of LBT and PPTN on levels of extracellular DNA in MSU-treated neutrophils. All values are presented as the mean ± SD. ($^{\#}P < 0.05$, $^{\#\#}P < 0.01$, $^{\#\#\#}P < 0.001$

versus corresponding Control. $^{*}P<0.05$; $^{**}P<0.01$; $^{***}P<0.001$ versus Model group, One–Way ANOVA). For **B** to **C**, WT rats were intra-articularly injected with 5 mg/ml MSU to induce gouty arthritis. Rats were additionally treated with Lobetyolin (10 μM) and PPTN (10μM) or Veh respectively as indicated. **B** Western blot analysis and relative quantification of NETosis-related proteins in synovial tissues. **C** Western blot analysis and relative quantification of apoptosis-related proteins in synovial tissues. For **D** to **E**, WT rats were injected with 5 mg/ml MSU crystals into pre-formed air pouches on the backs. Rats were additionally treated with LBT (10 μM), PPTN (10 μM) or Veh respectively as indicated. **D** Western blot analysis and relative quantification of NETosis-related proteins in air pouch neutrophils. **E** Western blot analysis and relative quantification of apoptosis-related proteins in air pouch neutrophils. For **F** to **G**, Primary Neutrophils were prepared from the bone marrow of WT rats and then treated with 250 μg/ml MSU. Neutrophils were additionally incubated with LBT (10 μM), PPTN (10 μM) or Veh respectively as indicated. **F** Western blot analysis and relative quantification of NETosis-related proteins in primary neutrophils. Quantification of the protein level of IL-1β and IL-8 in supernatant was expressed as the ratio of WT+Vehicle group. **G** Western blot analysis and relative quantification of apoptosis-related proteins in primary neutrophils. All values are presented as the mean ± SD. ($^{\#}P < 0.05$, $^{\#\#}P < 0.01$, $^{\#\#\#}P < 0.001$ versus corresponding WT+Vehicle group. $^{*}P<0.05$; $^{**}P<0.01$; $^{***}P<0.001$ versus corresponding WT+MSU group, One–Way ANOVA).

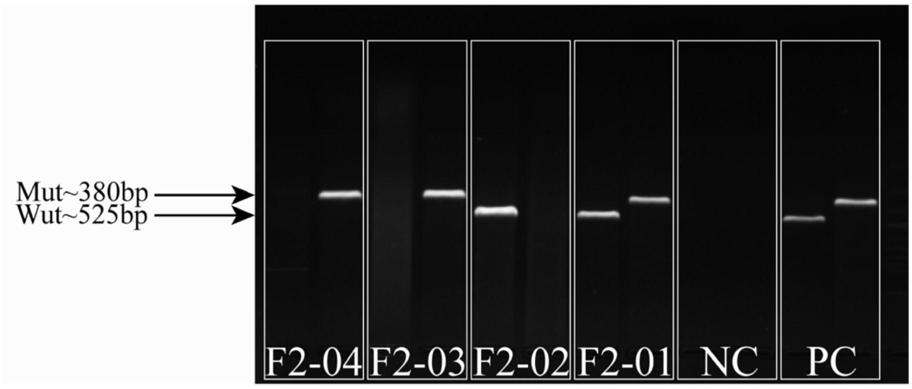

Supplementary Fig. 7 Representative gene identification results of F2 generation rats. PC represents a positive control and NC represents a negative control.

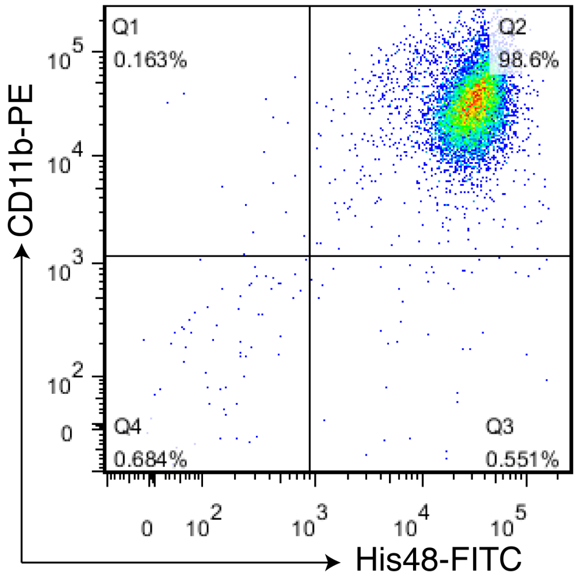

Supplementary Fig. 8 Representative FACS images of CD11b/Hs48-stained neutrophils purified using RBC lysis and Histopaque methods.

Supplementary Table

Supplementary Table 1. Reagents and antibodies

Reagent		
Reagent	Company	Cat. No
IBMX	Sigma-Aldrich	I7018
Ro 20-1724		B8279
Uric acid sodium salt		U2875
Histopaque-1119		11191
Histopaque-10771		10771
Forskolin	MCE	HY-15371
SQ22536		HY-100396
Bucladesine		HY-B0764
H-89		HY-15979
cAMP-Glo ™ Assay	Promega	V1502
In Situ Cell Death Detection Kit	Roche	11684817910
SYTOX Green Nucleic Acid Stain	Thermo Fisher	S7020
Lobetyolin	Topscience	T3825
All-In-One RT Master Mix Kit	ABM	G490
AceQ Universal SYBR qPCR Master Mix	Vazyme	Q511-02
Antibody		
Mouse anti-MPO	Abcam	ab90810
rabbit anti-cit-H3		ab5103

Goat anti-Mouse IgG H&L (Alexa Flour® 568)		ab175473
Donkey Anti-Rabbit IgG H&L (Alexa Flour® 647)		ab150075
Monoclonal Antibody (HIS48)-FITC	eBioscience	11-0570-82
CD11b/c Monoclonal Antibody (OX42)-PE		12-0110-82
rabbit anti-Phospho-PKA alpha+beta	BIOSS	bs-3725R
rabbit anti-Phospho-Raf1(Ser338)		bs-3377R
rabbit anti-Phospho-MER1/2(Ser218+Ser222)		bs-3270R
rabbit anti-Erk1/2		bs-0022R
rabbit anti-Phospho-ERK1/2 (Thr202+Tyr204)		bs-3016R
rabbit anti-NOX2		bs-3889R
rabbit anti-PI3K		bs-3332R
rabbit anti-Phospho-PI3K		bs-3332R
rabbit anti-Akt1+2+3		bs-6951R
rabbit anti-Phospho-Akt (Ser473)		bs-0876R
rabbit anti-Bcl-2		bs-4563R
rabbit anti-Bax		bs-0127R
rabbit anti-IL-1Beta		bs-6319R
rabbit anti-β-actin		bs-0061R
goat anti- rabbit IgG HRP		bs-0295G
rabbit anti-IL-8	Affinity	DF-6998

Supplementary Table 2. Primers

Primers for Standard polymerase chain reaction		
Gene	Forward (5’ to 3’)	Reverse (5’ to 3’)
GPR105-WT	CTTTGTAACCACTATGCCCAAGCAG	TGATTTGACTTAAGGCCCAGCAGTA
GPR105-Mut	AGGCAGGAGGAAGTGTCAGGT	GCTCAGAGCCTTGCTTTTTGGTG
Primers for Real-time PCR		
hGPR105	TACGTGCCCAGCTCTAAGAGT	GTCACCAAGGATCTTGAAAGGAA
hGADPH	ATCCCATCACCATCTTCCAGG	GATGACCCTTTTGGCTCCC
